# Comparative efficacy of two novel bioceramic root canal sealers in the treatment of chronic apical periodontitis

**DOI:** 10.1097/MD.0000000000050962

**Published:** 2026-10-16

**Authors:** Jiahuan He, Liye Song, Bingkun Cheng, Yue Cui, Fenjiao Liu, Xue Song, Yingqi Liu

**Affiliations:** a General Dentistry Department, Handan Central Hospital, Handan City, Heibei Province, China; b Department of Stomatology, Affiliated Hospital of Hebei University of Engineering, Handan City, Heibei Province, China; c Department of Oral and Maxillofacial Surgery, Handan Central Hospital, Handan City, Heibei Province, China.

**Keywords:** bioceramic sealer, calcium silicate–based sealer, chronic apical periodontitis, radiographic healing, root canal therapy

## Abstract

Chronic apical periodontitis (CAP) is a persistent periradicular inflammatory condition driven by intraradicular infection and remains a common indication for primary nonsurgical root canal therapy. This retrospective cohort study compared 2 calcium silicate-based bioceramic root canal sealers (C-Root SP vs iRoot SP) used during obturation in adults treated for CAP between January 2022 and December 2024. CAP was confirmed by compatible clinical findings and radiographic evidence of periapical radiolucency; cone-beam computed tomography (CBCT) was obtained when clinically indicated. The primary endpoint was a strict composite treatment success endpoint requiring both clinical resolution and radiographic healing at final follow-up. Secondary outcomes included pain severity measured by the visual analog scale (VAS) and treatment-related events. Among 227 eligible patients, 209 were included in the final analysis (C-Root SP, n = 101; iRoot SP, n = 108). Treatment success was higher with C-Root SP than with iRoot SP (86.1% vs 74.1%; χ^2^ = 4.73, *P* = .030), and radiographic healing was also more frequent (88.1% vs 76.9%; χ^2^ = 4.55, *P* = .033), whereas clinical resolution did not differ statistically (91.1% vs 83.3%; *P* = .095). After adjustment for age, sex, baseline lesion diameter, tooth location, diabetes mellitus, and baseline sinus tract, C-Root SP was associated with higher odds of treatment success (adjusted odds ratio [aOR] = 2.25, 95% confidence interval [CI]: 1.09–4.65; *P* = .028) and radiographic healing (aOR = 2.23, 95% CI: 1.04–4.79; *P* = .040), while the adjusted association with clinical resolution was not statistically significant (aOR = 2.12, 95% CI: 0.89–5.09; *P* = .091). VAS trajectories were similar overall, with a modest between-group difference at 1 month. Postoperative pain, acute exacerbations, sealer extrusion, and further endodontic interventions were infrequent and comparable between groups. In exploratory analysis, sealer extrusion was associated with treatment failure (OR = 4.91, *P* = .002). These observational findings indicate associations rather than causal treatment effects and require prospective confirmation.

## 1. Introduction

Chronic apical periodontitis (CAP) represents a persistent periradicular inflammatory disease primarily sustained by intraradicular microbial biofilms and their byproducts, and it remains a common indication for primary nonsurgical root canal therapy. Contemporary endodontic management aims to achieve durable infection control through chemo-mechanical preparation and irrigation, followed by obturation that limits coronal and apical leakage and supports periapical tissue repair. Despite high overall success rates, incomplete radiographic healing and late persistence of apical radiolucency remain clinically relevant, particularly because radiographic resolution may lag behind symptom improvement and because follow-up imaging can reveal asymptomatic nonhealing.^[1,2]^

Root canal sealers are integral to obturation because they occupy the sealer–dentin interface, penetrate dentinal tubules to varying degrees, and contribute to the resistance of the filled canal system to fluid movement and microbial ingress. Calcium silicate–based (bioceramic) sealers have been increasingly adopted due to their hydraulic setting reactions, favorable biocompatibility profiles, and potential bioactivity at the dentin interface. Recent clinical evidence indicates that calcium silicate–based sealer techniques can achieve clinical and radiographic outcomes comparable to established materials, although the certainty of evidence is limited by heterogeneity in obturation approaches, imaging modalities, and endpoint definitions.^[3,4]^ Pain and short-term functional recovery are additional considerations for patients undergoing root canal therapy. Systematic evidence syntheses published in the last 3 years suggest that postoperative pain following obturation with bioceramic sealers is generally comparable to resin-based sealers, with only modest differences reported across studies and follow-up windows.^[5,6]^ Treatment-related events such as sealer extrusion are clinically relevant because they may reflect apical anatomy, working length control, or obturation dynamics, and they have been examined as potential contributors to postoperative symptoms and delayed healing in contemporary clinical investigations.^[7]^

Among commercially available bioceramic sealers, iRoot SP has been evaluated in clinical and translational studies, including investigations linking sealer use to periodontal parameters and functional outcomes in patients with CAP and laboratory assessments of biocompatibility, penetrability, sealing ability, and antibacterial performance.^[8,9]^ However, comparative evidence across distinct bioceramic formulations remains incomplete, and direct head-to-head evaluations using standardized treatment protocols and clinically meaningful composite endpoints that integrate symptom resolution with radiographic healing are still needed to inform material selection in routine practice. Against this background, the present retrospective cohort study compared 2 bioceramic root canal sealers (C-Root SP and iRoot SP) in adults treated for CAP, assessing overall treatment success and treatment-related outcomes under a consistent clinical protocol.

## 2. Materials and methods

### 2.1. Study design

This study was approved by the Ethics Committee of Handan Central Hospital. This retrospective cohort study consecutively included adult patients (≥18 years) with a diagnosis of chronic apical periodontitis who underwent primary nonsurgical root canal therapy at our institution between January 2022 and December 2024. Patients were classified according to the bioceramic root canal sealer used for obturation into a C-Root SP group and an iRoot SP group. Chronic apical periodontitis was confirmed by compatible clinical findings and radiographic evidence of periapical radiolucency, and only cases with complete baseline documentation and at least 1 posttreatment follow-up evaluation were eligible. Patients were excluded if they presented with acute apical abscess or other acute endodontic infection requiring urgent drainage; had received prior endodontic treatment of the index tooth, including retreatment or apical surgery; exhibited perforation or other iatrogenic complications affecting the index tooth; had periapical pathology primarily attributable to periodontal disease or advanced periodontitis with a primary periodontal lesion; had root fracture, internal or external root resorption, or teeth deemed non-restorable; had systemic conditions expected to materially interfere with periapical healing, including uncontrolled diabetes mellitus, immunodeficiency, ongoing chemotherapy, or long-term systemic corticosteroid or other immunosuppressive therapy; were pregnant or lactating; or had incomplete clinical or radiographic data or were lost to follow-up. The study was conducted in accordance with the Declaration of Helsinki and was approved by the institutional medical ethics committee; written informed consent was obtained from all participants, and all analyses were performed using de-identified data to protect patient confidentiality.

### 2.2. Diagnostic criteria and definitions

Chronic apical periodontitis (CAP) was diagnosed based on a combination of clinical assessment and radiographic evidence. Clinically, CAP was suspected in teeth presenting with 1 or more of the following findings: percussion tenderness and/or occlusal discomfort, localized apical tenderness on palpation, and the presence of a sinus tract without signs of acute swelling. Radiographic confirmation was defined as a periapical radiolucency and/or widening of the periodontal ligament space at the apex on periapical radiographs; cone-beam computed tomography (CBCT) was used when additional 3-dimensional assessment was clinically indicated.

Treatment outcome was determined at follow-up using predefined criteria. Treatment success was defined as the absence of persistent or recurrent clinical signs or symptoms (including pain on percussion/occlusion, apical tenderness, or sinus tract) together with radiographic healing, indicated by resolution or a clear reduction in the size of the periapical radiolucency compared with baseline. Treatment failure was defined, for the prespecified composite endpoint, as failure to meet both components of treatment success, including persistent or recurrent clinical symptoms/signs, radiographic nonhealing or progression (unchanged or enlarged radiolucency), or the need for further endodontic intervention, apical surgery, or extraction attributable to ongoing apical disease. Because radiographic resolution can lag behind symptom resolution, asymptomatic cases with persistent radiographic abnormalities were additionally described by their clinical and radiographic components and were not interpreted as unequivocal biological failure in Section 4.

### 2.3. Interventions and standardized root canal treatment protocol

All procedures were performed by the same endodontist or by a fixed team of experienced endodontists following a standardized clinical protocol. Local anesthesia was administered when indicated, and all teeth were isolated with a rubber dam throughout treatment. After access cavity preparation, working length was determined using an electronic apex locator and verified radiographically with periapical imaging; cone-beam computed tomography (CBCT) was used for confirmation when clinically required.

Root canal preparation was completed using a standardized instrumentation approach with hand files and/or nickel–titanium rotary systems. Canals were enlarged to a predefined apical size and taper according to canal anatomy and clinical judgment, with copious irrigation applied between instrumentation steps using a standardized irrigant regimen. Following canal preparation, obturation was performed using a uniform filling technique, including a matched single-cone, cold lateral compaction, or warm gutta-percha technique as appropriate, with the root canal sealer applied according to the manufacturer’s instructions. In the C-Root SP group, canals were obturated using the bioceramic sealer C-Root SP, whereas in the iRoot SP group, iRoot SP was used as the sealer. Coronal sealing was completed immediately after obturation to minimize microleakage.

### 2.4. Outcome measures

The primary endpoint was the overall treatment success rate at final follow-up, defined by a strict composite of clinical resolution and radiographic healing. Clinical resolution was defined as the absence of persistent or recurrent endodontic symptoms and signs, including spontaneous pain, pain on percussion or occlusion, apical tenderness on palpation, and the presence of a sinus tract. Radiographic healing was defined as complete resolution or a clear reduction in the size of the baseline periapical radiolucency on follow-up periapical radiographs (and CBCT when available), without evidence of new or progressive periapical bone destruction. The same predefined radiographic criteria were applied to both treatment groups. Cases not meeting both components were classified as treatment failure for the composite analysis. To address the known temporal lag between clinical improvement and radiographic repair, the clinical and radiographic components were also reported separately, and asymptomatic radiographic nonhealing was explicitly distinguished in the interpretation of results.

Secondary outcomes included clinical symptom measures and treatment-related events. Clinical symptoms comprised patient-reported pain severity assessed using a visual analog scale (VAS), as well as clinician-assessed percussion tenderness, occlusal pain, and sinus tract recurrence during follow-up. Treatment-related events included postoperative pain, acute exacerbation requiring unscheduled visits or additional medication/intervention, radiographically evident sealer extrusion beyond the apex, and the need for further endodontic management, including retreatment, apical surgery, or extraction attributable to persistent apical disease.

### 2.5. Baseline variables and potential confounders

Baseline data were extracted from electronic medical records and standardized endodontic charts to evaluate group comparability and potential confounding. Patient-level variables included age, sex, smoking status, and diabetes mellitus. Tooth-level variables comprised tooth type/location, number of root canals, baseline periapical lesion size, preoperative symptoms, and sinus tract. Treatment-level variables included single- versus multiple-visit treatment, intracanal medicament use, obturation technique, definitive coronal restoration, and timing of definitive restoration. Follow-up duration was additionally treated as an outcome-relevant covariate because radiographic healing is time-dependent. Group balance was assessed using both conventional hypothesis tests and standardized mean differences (SMDs); an absolute SMD < 0.10 was considered indicative of negligible imbalance. These variables informed the prespecified adjusted analysis described below.

### 2.6. Statistical analysis

All statistical analyses were performed using IBM SPSS Statistics, version 27.0 (IBM Corp., Armonk). Continuous variables are presented as mean ± standard deviation (SD) when approximately normally distributed and were compared between groups using the independent-samples *t*-test. Continuous variables with non-normal distributions are presented as median (interquartile range [IQR]) and were compared using the Mann–Whitney *U* test. Categorical variables are summarized as counts (percentages) and were compared using the chi-square test; Fisher exact test was applied when expected cell counts were small. Associations between categorical variables, including the relationship between sealer extrusion and composite nonsuccess, were evaluated using odds ratios (ORs) with corresponding 2-sided *P* values. All tests were 2-tailed, and *P* < .05 was considered statistically significant.

## 3. Results

### 3.1. Patient enrollment and case selection

Between January 2022 and December 2024, a total of 227 adult patients diagnosed with chronic apical periodontitis (CAP) underwent primary nonsurgical root canal therapy at our institution and were assessed for eligibility. After sequential application of the predefined inclusion and exclusion criteria, 18 patients were excluded for the following reasons: acute apical abscess or acute endodontic infection requiring urgent drainage (n = 5), history of prior endodontic treatment of the index tooth including retreatment or apical surgery (n = 4), periapical pathology primarily attributable to periodontal disease (n = 3), root fracture or internal/external root resorption (n = 2), systemic conditions expected to materially interfere with periapical healing (n = 2), and incomplete clinical or radiographic records or loss to follow-up (n = 2). Consequently, 209 patients were included in the final analysis, comprising 101 in the C-Root SP group and 108 in the iRoot SP group. The unit of analysis was the patient, and 1 index tooth per patient was evaluated, yielding 209 treated teeth included in the outcome assessment.

### 3.2. Baseline characteristics and group comparability

At baseline, the C-Root SP group (n = 101) and the iRoot SP group (n = 108) were comparable across patient-, tooth-, and treatment-level characteristics (Table 1). No statistically significant between-group differences were observed in age (*P* = .517), sex distribution (*P* = .439), smoking status (*P* = .829), or diabetes mellitus prevalence (*P* = .167). Tooth-level variables, including posterior tooth distribution (*P* = .160), number of canals (*P* = .336), baseline lesion diameter (*P* = .357), preoperative pain (*P* = .562), percussion/occlusal tenderness (*P* = .384), and sinus tract prevalence (*P* = .387), were also balanced. At the treatment-process level, the proportions of single-visit treatment (57.4% vs 55.6%, *P* = .894) and intracanal medicament use (*P* = .898) did not differ significantly, and obturation technique distribution (*P* = .155), definitive restoration type (*P* = .151), and timely definitive restoration within 7 days (*P* = .443) were similar between groups.

**Table 1:** Baseline characteristics of patients and treated teeth in the C-Root SP and iRoot SP groups.

Variable	Category	C-Root SP (n = 101)	iRoot SP (n = 108)	*P* value	Absolute SMD
Age, yr		43.59 ± 12.21	44.70 ± 11.51	0.500	0.094
Sex	Female	55 (54.5%)	52 (48.1%)	0.439	0.126
Sex	Male	46 (45.5%)	56 (51.9%)		0.126
Smoking status	Never	59 (58.4%)	65 (60.2%)	0.829	0.036
Smoking status	Current	18 (17.8%)	21 (19.4%)		0.042
Smoking status	Former	24 (23.8%)	22 (20.4%)		0.082
Diabetes mellitus	Yes	12 (11.9%)	6 (5.6%)	0.167	0.226
Tooth location	Posterior	51 (50.5%)	66 (61.1%)	0.160	0.215
Number of canals	1	32 (31.7%)	25 (23.1%)	0.336	0.192
Number of canals	2	46 (45.5%)	52 (48.1%)		0.052
Number of canals	3+	23 (22.8%)	31 (28.7%)		0.136
Baseline lesion diameter, mm		5.05 ± 2.75	5.23 ± 2.69	0.633	0.066
Preoperative pain	Yes	39 (38.6%)	47 (43.5%)	0.562	0.100
Percussion/occlusal tenderness	Yes	51 (50.5%)	47 (43.5%)	0.384	0.140
Baseline sinus tract	Yes	10 (9.9%)	16 (14.8%)	0.387	0.150
Treatment visits	Single visit	58 (57.4%)	60 (55.6%)	0.894	0.038
Intracanal medicament	Yes	43 (42.6%)	44 (40.7%)	0.898	0.037
Obturation technique	Matched single cone	51 (50.5%)	54 (50.0%)	0.408	0.010

Absolute SMDs are shown; values ≥0.10 indicate potentially relevant imbalance. *P* values are descriptive and were not used as the sole criterion for covariate balance.

SMD = standardized mean difference.

### 3.3. Primary outcome: overall treatment success

At the final follow-up, the overall composite treatment success rate was 86.1% (87/101) in the C-Root SP group and 74.1% (80/108) in the iRoot SP group, demonstrating a statistically significant between-group difference (χ^2^ = 4.73, *P* = .030). When the composite endpoint was decomposed, clinical resolution was observed in 91.1% of patients in the C-Root SP group and 83.3% in the iRoot SP group (*P* = .095), whereas radiographic healing occurred in 88.1% and 76.9%, respectively (χ^2^ = 4.55, *P* = .033; Table 2). Among patients who did not meet the composite success criteria, the most common pattern was radiographic nonhealing or progression without persistent symptoms (50.0% in both groups), followed by persistent or recurrent clinical symptoms without radiographic progression (28.6% in both groups).

**Table 2:** Primary outcome and its component endpoints at final follow-up.

Outcome	C-Root SP (n = 101)	iRoot SP (n = 108)	Test statistic	*P* value
Overall treatment success (composite)	87/101 (86.1)	80/108 (74.1)	χ^2^ = 4.73	.030
Clinical resolution	92/101 (91.1)	90/108 (83.3)	χ^2^ = 2.79	.095
Radiographic healing	89/101 (88.1)	83/108 (76.9)	χ^2^ = 4.55	.033

χ^2^ = chi-square statistic.

### 3.4. Secondary outcomes: symptoms and clinical findings

Baseline VAS scores were comparable between the C-Root SP and iRoot SP groups (4.32 ± 1.63 vs 4.40 ± 1.70, *P* = .721), and no between-group difference was observed at 1 week (2.36 ± 1.34 vs 2.45 ± 1.31, *P* = .621). At 1 month, the C-Root SP group reported a lower mean VAS score than the iRoot SP group (0.89 ± 0.75 vs 1.14 ± 0.90, *P* = .029). VAS scores remained low at final follow-up (0.46 ± 0.52 vs 0.60 ± 0.55), with no statistically significant difference between groups (*P* = .056). The overall reduction in pain from baseline to final follow-up was similar between groups (change in VAS: -3.86 ± 1.66 vs -3.80 ± 1.87, *P* = .808). At final follow-up, the frequencies of residual clinical signs and symptom events were low and did not differ significantly between groups. Percussion tenderness positivity was observed in 5.9% of patients in the C-Root SP group and 9.3% in the iRoot SP group (*P* = .367), occlusal pain occurred in 4.0% and 7.4%, respectively (*P* = .284), and sinus tract recurrence was documented in 2.0% and 3.7%, respectively (Fisher exact test, *P* = .684; Table 3).

**Table 3:** Secondary outcomes: pain intensity over time and clinical signs at final follow-up.

Outcome	C-Root SP (n = 101)	iRoot SP (n = 108)	Test statistic	*P* value
Pain intensity (VAS), mean ± SD				
VAS at baseline (T0)	4.32 ± 1.63	4.40 ± 1.70	*t* = −0.36	.721
VAS at 1 wk (T1)	2.36 ± 1.34	2.45 ± 1.31	*t* = −0.50	.621
VAS at 1 mo (T2)	0.89 ± 0.75	1.14 ± 0.90	*t* = −2.20	.029
VAS at final follow-up (T3)	0.46 ± 0.52	0.60 ± 0.55	*t* = −1.92	.056
Change in VAS (T3–T0)	−3.86 ± 1.66	-3.80 ± 1.87	*t* = −0.24	.808
Clinical signs/symptoms at final follow-up, n (%)				
Percussion tenderness positive	6 (5.9)	10 (9.3)	χ^2^ = 0.81	.367
Occlusal pain	4 (4.0)	8 (7.4)	χ^2^ = 1.15	.284
Sinus tract recurrence	2 (2.0)	4 (3.7)	OR = 0.53	.684

OR = odds ratio, SD = standard deviation, *t* = Student *t* statistic, VAS = visual analog scale, χ^2^ = chi-square statistic.

### 3.5. Complications and treatment-related events

The incidence of postoperative pain within 72 hours was 27.7% in the C-Root SP group and 31.5% in the iRoot SP group, with no statistically significant difference between groups (χ^2^ = 0.35, *P* = .552). Among patients reporting postoperative pain, severity distribution was similar between groups (*P* = .899), and analgesic use within 72 hours did not differ significantly (17.8% vs 20.4%, *P* = .640). Acute exacerbation events requiring unscheduled visits or additional medication/intervention were infrequent and comparable between groups (5.9% vs 9.3%, *P* = .367). Radiographic sealer extrusion occurred in 8.9% of the C-Root SP group and 10.2% of the iRoot SP group (*P* = .754). Further endodontic management was uncommon, including retreatment (2.0% vs 3.7%, *P* = .684), apical surgery (1.0% vs 1.9%, *P* = 1.000), and extraction (1.0% vs 1.9%, *P* = 1.000). In an exploratory pooled analysis, sealer extrusion was associated with an increased likelihood of treatment failure (OR = 4.91, *P* = .002), and this association was directionally consistent within each treatment group (Table 4).

**Table 4:** Complications and treatment-related events during follow-up.

Outcome	C-Root SP (n = 101)	iRoot SP (n = 108)	Test statistic	*P* value
Postoperative pain within 72 h, n (%)	28 (27.7)	34 (31.5)	χ^2^ = 0.35	.552
Pain severity among those with pain, n (%)	Mild 20 (71.4); Moderate 7 (25.0); Severe 1 (3.6)	Mild 23 (67.6); Moderate 9 (26.5); Severe 2 (5.9)	χ^2^ = 0.21	.899
Analgesic use within 72 h, n (%)	18 (17.8)	22 (20.4)	χ^2^ = 0.22	.640
Acute exacerbation/unscheduled visit, n (%)	6 (5.9)	10 (9.3)	χ^2^ = 0.81	.367
Radiographic sealer extrusion, n (%)	9 (8.9)	11 (10.2)	χ^2^ = 0.10	.754
Endodontic retreatment, n (%)	2 (2.0)	4 (3.7)	OR = 0.53	.684
Apical surgery, n (%)	1 (1.0)	2 (1.9)	OR = 0.53	1.000
Tooth extraction, n (%)	1 (1.0)	2 (1.9)	OR = 0.53	1.000

OR = odds ratio, χ^2^ = chi-square statistic.

## 4. Discussion

This retrospective cohort study compared 2 bioceramic root canal sealers (C-Root SP and iRoot SP) used during primary nonsurgical root canal therapy for chronic apical periodontitis. The principal finding was a higher composite treatment success rate at final follow-up in the C-Root SP group than in the iRoot SP group (86.1% vs 74.1%), with the between-group difference reaching statistical significance. When the composite endpoint was decomposed, the intergroup separation was driven primarily by radiographic healing (88.1% vs 76.9%), whereas clinical resolution demonstrated a smaller, nonsignificant difference. This pattern – greater divergence in radiographic outcome than in symptom resolution – is clinically plausible in chronic apical periodontitis, in which symptoms often abate earlier than complete periapical bone repair, and radiographic improvement may lag behind clinical recovery. Contemporary outcome frameworks and guidelines emphasize the need to integrate clinical and radiographic criteria when defining endodontic success, particularly for apical disease.^[10,11]^

The higher observed treatment success and radiographic healing proportions in the C-Root SP group indicate an association between sealer type and outcome within this cohort; they should not be interpreted as evidence that C-Root SP itself caused superior healing. Although measured baseline factors were assessed with SMDs, and the primary comparison was additionally adjusted for prespecified confounders, residual confounding remains possible because treatment allocation was not randomized. The radiographic difference could reflect material-related factors, but it could also be influenced by case selection, follow-up duration, anatomical complexity, or other unmeasured clinical factors. Therefore, any mechanistic interpretation remains hypothesis-generating.^[12,13]^

The present findings align with emerging clinical evidence that calcium silicate–based sealers can achieve favorable clinical and radiographic outcomes when used with contemporary obturation techniques. A systematic review and meta-analysis comparing bioceramic single-cone obturation with conventional approaches reported broadly comparable clinical outcomes overall, while highlighting heterogeneity in included studies and outcome definitions.^[14]^ In addition, clinical comparative studies evaluating resin-based versus bioceramic sealers have reported that bioceramic approaches can perform similarly or, in some contexts, show advantages on selected outcomes, although the direction and magnitude of effect are not uniform across materials and protocols.^[15]^ Against this background, the higher radiographic healing proportion observed with C-Root SP may reflect differences in handling properties, flow, setting characteristics, or interaction with dentin and periapical tissues between the 2 bioceramic sealers. Because the current analysis did not directly quantify sealer physicochemical behavior or interfacial adaptation, the mechanistic explanation remains inferential and should be interpreted as hypothesis-generating.

Clinical resolution rates were high in both groups, and residual symptom events at final follow-up were uncommon. This is consistent with the general expectation that effective chemo-mechanical preparation, irrigation, and adequate coronal sealing will eliminate or reduce symptoms for most patients with chronic apical disease. The nonsignificant difference in clinical resolution, despite significant separation in radiographic healing, may indicate that sealer-related effects manifest more clearly on tissue-level repair than on short- to medium-term symptom control. Alternatively, clinical symptom metrics can exhibit lower sensitivity than radiographic measures in chronic apical periodontitis because many cases are minimally symptomatic at baseline or become asymptomatic early after treatment. Recent work evaluating radiographic outcomes commonly uses structured criteria, frequently anchored to indices such as the Periapical Index (PAI), while also noting that imaging modality influences outcome ascertainment; cone-beam computed tomography can identify persistent lesions not visible on periapical radiographs.^[16,17]^ Accordingly, the extent to which the observed radiographic advantage would persist under uniform CBCT-based assessment warrants further study.

Pain intensity decreased substantially in both groups from baseline to final follow-up, and the overall magnitude of pain reduction was similar. The transient difference at 1 month, with lower mean VAS in the C-Root SP group, should be interpreted cautiously. Although statistically significant, the absolute between-group difference in mean VAS at that time point was small, and the clinical relevance depends on prespecified minimal clinically important difference thresholds, which were not defined in this study. The broader pattern – similar early postoperative pain incidence and comparable analgesic utilization – aligns with recent evidence syntheses suggesting that postoperative pain is often similar when comparing bioceramic sealers with traditional sealers, particularly when instrumentation, irrigation, and obturation protocols are standardized.^[5,18]^ The low rates of percussion tenderness, occlusal pain, and sinus tract recurrence at final follow-up further suggest that both sealers were compatible with effective symptom control under the applied clinical protocol.

Adverse events and treatment-related sequelae were infrequent in both groups, including acute exacerbations requiring unscheduled visits and the need for further endodontic interventions (retreatment, apical surgery, extraction). Radiographic sealer extrusion occurred in approximately 9% to 10% of cases and did not differ between groups. This extrusion rate is within the range reported in contemporary clinical studies, though rates vary depending on obturation technique, apical anatomy, patency management, and sealer rheology.^[7,19]^ The exploratory analysis indicated an association between sealer extrusion and composite nonsuccess. This observation is biologically plausible because extrusion may reflect over-instrumentation, larger apical foramina, or complex apical anatomy, each of which could be linked to persistent periapical inflammation or delayed healing. Additionally, extruded material could contribute to local tissue irritation, depending on its biocompatibility and the volume extruded. However, the literature is mixed regarding the clinical impact of extrusion, with some studies identifying associations with pain or adverse outcomes under particular conditions and others reporting minimal clinical consequence when extrusion volumes are small and materials are biocompatible.^[20,21]^ Given the retrospective design and limited number of failures, the extrusion–nonsuccess association in this cohort should be treated as an exploratory signal rather than definitive evidence of causality.

From a practice perspective, C-Root SP was associated with a higher probability of meeting the composite success criterion, primarily because of a higher proportion of radiographic healing. However, given the retrospective observational design, this association should not be interpreted as evidence of causality or as support for preferential clinical selection of C-Root SP. Both sealers were associated with favorable symptom trajectories and low rates of adverse events, and sealer choice should therefore be considered as only 1 component of a broader treatment strategy that also includes effective canal disinfection, appropriate obturation, and adequate coronal sealing, as emphasized in contemporary endodontic guidelines and outcome frameworks.^[22]^

The predominance of asymptomatic radiographic nonhealing among treatment-failure cases also highlights an important limitation in outcome interpretation. Because radiographic repair may lag behind clinical symptom resolution, assessment at the available final follow-up may classify some clinically asymptomatic teeth as treatment failures despite ongoing periapical healing. Accordingly, the observed difference in radiographic healing should be interpreted cautiously. Prospective studies with standardized imaging protocols, predefined follow-up intervals, and longer-term assessment are needed to determine whether this association persists over time.

Several limitations should be considered. First, the retrospective design introduces susceptibility to selection bias, information bias, and residual confounding. Although baseline characteristics were balanced, unmeasured variables – such as lesion chronicity, microbial load, canal morphology complexity, operator micro-variations, and quality of coronal restoration – may influence outcomes. Second, the follow-up schedule and imaging modality were not necessarily uniform across all cases, and radiographic healing assessment using periapical radiographs may under-detect persistent lesions compared with CBCT. Third, the composite endpoint – while clinically meaningful – combines clinical and radiographic components that may change at different rates; this can complicate interpretation of “nonsuccess” cases that are asymptomatic but show delayed radiographic repair. Fourth, the study did not quantify obturation quality metrics (length, density, voids) in a standardized manner, nor did it measure sealer extrusion volume or apical anatomy parameters, limiting mechanistic inference. Finally, single-center data may limit generalizability to settings with different operator experience or patient case mix.

## 5. Conclusion

In this retrospective cohort, C-Root SP was associated with higher radiographic healing and a higher composite treatment success rate than iRoot SP, while clinical symptom resolution and adverse-event profiles were broadly similar. Because treatment allocation was non-randomized and residual confounding and follow-up-related misclassification remain possible, these findings do not establish a causal advantage or support a definitive clinical recommendation. Prospective studies with standardized imaging intervals and adequate follow-up are needed to confirm this association.

## Author contributions

**Conceptualization:** Jiahuan He, Liye Song, Bingkun Cheng, Yue Cui, Fenjiao Liu, Xue Song, Yingqi Liu.

**Data curation:** Jiahuan He, Liye Song, Bingkun Cheng, Yue Cui, Fenjiao Liu, Xue Song, Yingqi Liu.

**Formal analysis:** Jiahuan He, Liye Song, Bingkun Cheng, Yue Cui, Fenjiao Liu, Xue Song, Yingqi Liu.

**Funding acquisition:** Yingqi Liu.

**Writing** – **original draft:** Yingqi Liu.

**Writing** – **review & editing:** Yingqi Liu.
